# Between Two Fern Genomes

**DOI:** 10.1186/2047-217X-3-15

**Published:** 2014-09-25

**Authors:** Emily B Sessa, Jo Ann Banks, Michael S Barker, Joshua P Der, Aaron M Duffy, Sean W Graham, Mitsuyasu Hasebe, Jane Langdale, Fay-Wei Li, D Blaine Marchant, Kathleen M Pryer, Carl J Rothfels, Stanley J Roux, Mari L Salmi, Erin M Sigel, Douglas E Soltis, Pamela S Soltis, Dennis W Stevenson, Paul G Wolf

**Affiliations:** 1 Department of Biology, Box 118525, University of Florida, Gainesville, FL 32611, USA; 2 Genetics Institute, University of Florida, Box 103610, Gainesville, FL 32611, USA; 3 Department of Botany and Plant Pathology, Purdue University, 915 West State Street, West Lafayette, IN 47907, USA; 4 Department of Ecology & Evolutionary Biology, University of Arizona, 1041 East Lowell Street, Tucson, AZ 85721, USA; 5 Department of Biology, Penn State University, 201 Life Science Building, University Park, PA 16801, USA; 6 Ecology Center and Department of Biology, Utah State University, 5305 Old Main Hill, Logan, UT 84322, USA; 7 Department of Botany, University of British Columbia, 3529-6720 University Blvd., Vancouver, BC V6T 1Z4, Canada; 8 National Institute for Basic Biology, 38 Nishigounaka, Myo-daiji-cho, Okazaki 444-8585, Japan; 9 Department of Plant Sciences, University of Oxford, South Parks Road, Oxford OX1 3RB, UK; 10 Department of Biology, Duke University, Post Office Box 90338, Durham, NC 27708, USA; 11 Florida Museum of Natural History, Dickinson Hall, University of Florida, Gainesville, FL 32611, USA; 12 Department of Zoology, University of British Columbia, 2329 W. Mall, WAITING Vancouver, BC V6T 1Z4, Canada; 13 Department of Molecular Biosciences, University of Texas, 205 W. 24th Street, Austin, TX 78712, USA; 14 New York Botanical Garden, 2900 Southern Boulevard, Bronx, NY 10458, USA; 15 Current address: Department of Biological Science, California State University, 800 N. State College Blvd., Fullerton, CA 92831, USA; 16 Current address: University Herbarium and Department of Integrative Biology, University of California, 1001 Valley Life Sciences Building, Berkeley, Berkeley, CA 94720, USA

**Keywords:** *Azolla*, *Ceratopteris*, Comparative analyses, Ferns, Genomics, Land plants, Monilophytes

## Abstract

Ferns are the only major lineage of vascular plants not represented by a sequenced nuclear genome. This lack of genome sequence information significantly impedes our ability to understand and reconstruct genome evolution not only in ferns, but across all land plants. *Azolla* and *Ceratopteris* are ideal and complementary candidates to be the first ferns to have their nuclear genomes sequenced. They differ dramatically in genome size, life history, and habit, and thus represent the immense diversity of extant ferns. Together, this pair of genomes will facilitate myriad large-scale comparative analyses across ferns and all land plants. Here we review the unique biological characteristics of ferns and describe a number of outstanding questions in plant biology that will benefit from the addition of ferns to the set of taxa with sequenced nuclear genomes. We explain why the fern clade is pivotal for understanding genome evolution across land plants, and we provide a rationale for how knowledge of fern genomes will enable progress in research beyond the ferns themselves.

## Introduction

Ferns (Monilophyta) are an ancient lineage of land plants that comprise a significant component of the Earth’s terrestrial flora. They are the second largest group of vascular plants, with more than 10,000 species [1], and play a major role in shaping community assembly and ecological processes in many biomes. For example, ferns shape ecosystem regeneration, persistence, and biomass production in eastern North American temperate forests [2-4]; play keystone roles in tropical rainforest canopies [5,6], heathlands [7], after landslides [8], and on islands [9]; and include several invasive species with significant economic impact [10-12]. Phylogenetically, ferns are sister to the seed plant clade (Spermatophyta) that includes the ecologically dominant flowering plants. Thus, the phylogenetic position of ferns makes them pivotal in the evolutionary history of land plants (Embryophyta), and essential for a comprehensive understanding of the origin and diversification of numerous traits found in seed plant crops and model species, such as rice and *Arabidopsis*[13,14].

## Review

**Figure 1:**
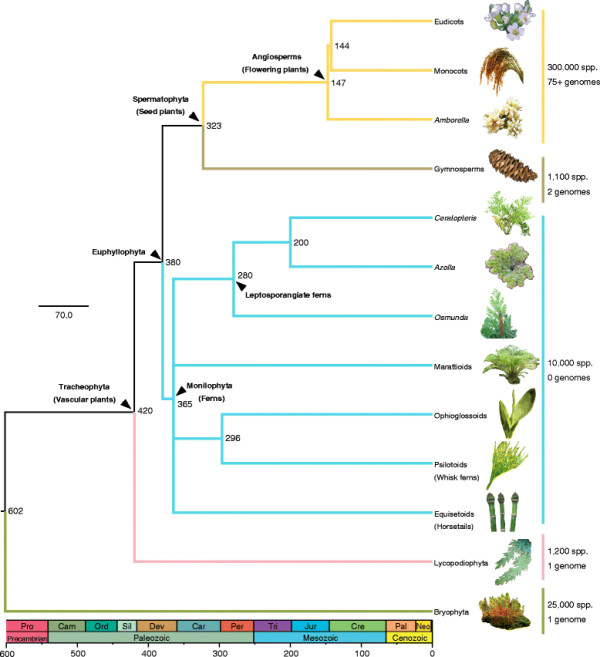
**Phylogeny of major groups of land plants.** Based on [13,15,19,20]. Approximate numbers of species and available genome sequences are given, and approximate times of major divergences are indicated. Ferns as a whole include lineages that diverged from one another prior to the divergence of the major seed plant clades. The most recent common ancestor of all leptosporangiates arose approximately 280 mya [17,18]. The ancestors of *Ceratopteris* and *Azolla* diverged from each other ca. 200 mya, well before the divergence of monocots and eudicots. Dates obtained from TimeTree [21,22].

In a broad sense, ferns include four main clades: psilotoids (whisk ferns) + ophioglossoids, equisetoids (horsetails), marattioids, and leptosporangiates (Figure 1). The leptosporangiate ferns are the most species-rich clade by far, with over 9,000 species [15,16] that include the majority of fern species found in temperate and tropical regions. Ferns and seed plants diverged from a common ancestor around 380 million years ago (mya) (the oldest fern fossils date to ca. 350 mya [17]), and the most recent common ancestor (MRCA) of the leptosporangiate ferns arose ca. 280 mya [17,18]. Several fern lineages diverged from one another prior to the divergence of the angiosperm and gymnosperm sister clades (Figure 1).

Despite the ubiquity of ferns and their ecological and evolutionary importance, genomic resources for the group remain sparse. Ferns are the only major clade of vascular land plants for which a complete nuclear genome has not yet been sequenced. This gap is particularly acute in light of recent efforts to sequence the transcriptomes of all major lineages of green plants [23,24]. The assembly, analysis, and interpretation of these transcriptomes would benefit enormously from the availability of well-annotated fern genomes. Recent innovations in sequencing technologies and the resulting torrent of whole-genome sequencing projects have fueled a renaissance in comparative genetic and genomic analyses, and each genome sequenced yields new insights into plant evolution. For example, the recently-sequenced genome of *Amborella trichopoda*[25]—the sister taxon to all other angiosperms—has revealed much about the conservation of synteny across flowering plants and about genome organization, as well as gene content in the ancestral angiosperm. It has also facilitated inference of ancient genome doubling events in angiosperms. Ferns, with their large genomes, high chromosome numbers, independent gametophyte phase, and mix of heterosporous and homosporous taxa, offer unparalleled opportunities for groundbreaking comparative genetic and genomic analyses across land plants as a whole.

Ferns provide a stark contrast to other lineages of land plants in several key biological features. For example, angiosperms and gymnosperms are both dominated by a diploid, spore-bearing (sporophyte) stage of the life cycle. Their haploid sexual stage, the gametophyte, is extremely reduced (microscopic in angiosperms) and completely dependent on the sporophyte for nutrition. On the other hand, in bryophytes (mosses, liverworts and hornworts), it is the sporophyte that is dependent at maturity on the dominant, macroscopic and photosynthetic gametophyte. Ferns and lycophytes are the only land plants where, for most taxa, both gametophytes and sporophytes are independent, free-living organisms that can each be long-lived. Unlike seed plants, which are exclusively heterosporous, ferns include both heterosporous and homosporous species. The latter group includes the majority of extant fern diversity, in which only one spore type is produced that develops into a gametophyte that is either bisexual, or whose sex is determined by non-genetic aspects of development (e.g., pheromones from surrounding gametophytes). The evolution from homospory to heterospory—in which a megaspore develops into a female gametophyte that includes one or more egg cells, and a microspore develops into a male gametophyte that includes sperm—is among the most important transitions in the evolution of plants, with profound effects on plant reproduction and the life cycle [26]. Nevertheless, the nuclear genome of a homosporous vascular plant has yet to be sequenced.

Cytological studies throughout the twentieth century revealed that ferns, especially homosporous species (which include up to 99% of extant ferns [1]), have significantly higher chromosome numbers than other plants [27-30]. Homosporous ferns average *n* = 57.05 chromosomes, compared to *n* = 15.99 for flowering plants [31], and the highest chromosome number known for any multicellular organism (*2n* = 1440) is that of the homosporous fern *Ophioglossum reticulatum*[32]. However, heterosporous ferns possess an average of only *n* = 13.62 chromosomes, very close to the average of flowering plants—another heterosporous lineage. To date, no explanatory hypothesis for this cross-lineage discrepancy in chromosome numbers vs. spore type has survived rigorous testing [33]. Along with their high chromosome numbers, many homosporous ferns have extremely large genomes [34-39], and homosporous ferns are the only land plants to show a strong positive correlation between chromosome number and genome size [40].

Because of their high chromosome numbers [41,42], homosporous ferns were initially assumed to have experienced many rounds of ancient whole-genome duplication (polyploidy) [31], events that have likely influenced the structures of all land plant genomes. In addition, two decades of experiments have consistently shown that homosporous ferns possessing the putative base chromosome numbers of their genus—even if those numbers are high compared to those of angiosperms—behave genetically as diploids (e.g., [43-52]). Ferns also lack chromosome-level evidence of extensive ancient polyploidy, such as syntenic chromosomal blocks [53,54]. This combination of high chromosome numbers and lack of evidence for extensive polyploidy in homosporous ferns has been referred to as the “polyploidy paradox” [55]. Whole-genome data are essential for resolving this paradox and also for understanding basic aspects of genome organization and different pathways for genome streamlining and diploidization—acting post-polyploidization—that may operate in ferns vs. angiosperms.

Paleopolyploidy events have been inferred in the histories of all angiosperm lineages studied to date (e.g., [56]) and are implicated in the ancestral angiosperm and ancestral seed plant genomes [25,57]. Thus, even contemporary flowering plant taxa with relatively small genomes, such as the model species *Arabidopsis thaliana* (*n* = 5, 125 Mb [58]), often belong to lineages that have experienced multiple rounds of polyploidy. *Arabidopsis* is thought to have experienced five such events, including the ancestral seed plant and angiosperm duplications [57,59]. Various groups have evidently responded to these events in different ways, and data from ferns are the key to understanding these differences. Using these data, we can ask, for example: how do the various genomic components (e.g., repetitive elements) differ across land plant lineages, and how do their fates differ following polyploidy? What mechanisms are responsible for the universally smaller numbers of chromosomes in heterosporous vs. homosporous lineages, and how do these relate to the transitions among mating systems across land plants? What genomic changes underlie trends in gametophyte reduction and the shift from haploid-dominant to diploid-dominant life cycles across land plants? Do the free-living, haploid gametophytes of ferns experience strong purifying selection? Ferns are the crucial missing clade for understanding all of these evolutionary paradoxes. Most importantly, the addition of ferns to the set of sequenced land plant genomes will also facilitate reconstruction of the ancestral euphyllophyte (ferns plus seed plants; Euphyllophyta) and vascular plant (Tracheophyta) genomes, and will inform efforts to reconstruct the ancestral seed plant genome by providing an outgroup that is more suitable for comparative analyses than are the currently available lycophyte [60] and moss [61] genomes. Improved understanding of genomic changes during the evolution of seed plants will provide a new perspective for examining key evolutionary innovations in that clade, such as the seed itself.

To capture and characterize the genetic, genomic, and ecological diversity of ferns, we recommend two candidates for genome sequencing: *Azolla* (Azollaceae: Salviniales) and *Ceratopteris* (Pteridaceae: Polypodiales). Both have been promoted as model ferns for genome sequencing [14,40,62,63] and together, *Azolla* and *Ceratopteris* are a powerful combination. They cumulatively represent more than 400 million years of independent evolution (MRCA 200 mya [16]), and embody the key genomic and life-history characteristics of interest for fern genome sequencing.

*Azolla* is a heterosporous, free-floating water fern with a compact, 750 Mb (1C) genome and *n* = 22 chromosomes [38,64]. It has long been valued in Southeast Asia as a green fertilizer due to its symbiotic relationship with *Nostoc azollae*, a cyanobacterium that lives in cavities enclosed by the leaf tissue of *Azolla*[65] and renders it capable of nitrogen fixation [66]. *Azolla* also has promise as a biofuel and bioremediator in carbon sequestration efforts [63]. In addition, *Azolla* has been implicated as the cause of a massive shift in Earth’s climate approximately 50 mya [67], when atmospheric carbon dioxide levels were apparently halved by *Azolla*-driven carbon sequestration [68-70]. A genome sequence for *Azolla* will allow us to explore its relationship with its symbionts and may facilitate efforts to harness its nitrogen-fixing ability on a scale large enough to provide an inexpensive source of nitrogen-rich fertilizer [71].

Recently, the BGI (formerly Beijing Genomics Institute) agreed to complete the first fern genome sequencing project, for *Azolla*, in collaboration with principal investigator K.M. Pryer and colleagues (see [72,73]). Supplemental funds were also raised through crowdfunding [74,75], and the PIs are currently gathering material for the project. This planned sequencing of *Azolla* will provide initial and much-needed genomic resources for ferns, but given the deep divergence times, variation in life-history characteristics, and diversity within this clade, one fern genome is simply not enough to address the full range of outstanding genomic questions in ferns and across land plants.

*Ceratopteris* provides an ideal contrast to *Azolla*. It is homosporous, and its genome is 11.26Gb (1C; DB Marchant, unpublished), an order of magnitude larger than that of *Azolla*. This size is more typical of genome sizes found in leptosporangiate ferns and is closer to the size scale of conifer genomes than to *Azolla. Ceratopteris* is the “*Arabidopsis* of the fern world”: it can be readily transformed with recombinant DNA [76,77] and has a fast life cycle, features that have made it an ideal genetic model system for studying sex expression and mating systems [78-81], spore and gametophyte development [82-87], and even plant responses to gravity during space flight [88]. In addition, a rapidly developing strain of *Ceratopteris* has been used extensively as an educational model system in undergraduate and K-12 biology instruction worldwide [89,90].

The earliest candidates for genome sequencing in plants tended to be those with small and simple genomes that could be assembled with relative ease. As the trend towards whole-genome sequencing intensifies, an increasing number of taxa with large or complex genomes will be of interest for complete nuclear genome sequencing. It is likely that most large fern genomes will not assemble easily using current techniques, making them important test cases for improved sequencing strategies, mapping, and especially assembly approaches, such as those recently developed for sequencing of the 22Gb (1C) loblolly pine [91,92] and 20Gb (1C) Norway spruce [93] genomes [94]. *Ceratopteris* will provide such an opportunity, and genetic resources for this species already exist to facilitate the assembly process. These include a genetic linkage map and mapping population comprising ~500 doubled haploid lines (DHLs) [53], which will allow efficient *de novo* sequencing and high-quality assembly, leveraging, for example, the recombinant population genome construction approach of Hahn *et al*. [95]. *Azolla* will provide a novel opportunity to sequence a plant nuclear genome that has co-evolved for more than 70 million years along with the genomes of its obligate, vertically-inherited symbiotic microbiome. The genome of one such symbiont has been sequenced [66], but additional components of the fern microbiome are not well characterized.

## Conclusions

Ferns are a phylogenetically pivotal and evolutionarily critical group of plants, yet they remain a group for which we lack extensive nuclear genomic resources. This is an astonishing reality, given the progress that has been made to date elsewhere across the tree of life. Transcriptome sequencing efforts such as the 1,000 Plants Project [23] have vastly expanded the gene sequence resources available for plants, but genes alone are insufficient to answer the most pressing questions in fern and land plant genome evolution. Ferns are crucial for understanding many aspects of plant development, physiology, metabolism, and evolution, and they hold the answers to key questions that have puzzled evolutionary and comparative biologists for more than a century. Between these two ferns—*Ceratopteris* and *Azolla—*evolution has operated for 400 million years, providing tremendous opportunity for differences to accumulate, both between these genomes and between ferns and other extant plants. Simultaneous sequencing of *Azolla* and *Ceratopteris* will close the phylogenetic gap in available plant genomes, and more importantly, will complete the critical framework necessary for rigorous comparative studies of genome structure and function across land plants.

## Abbreviations

Gb: Gigabases; Mb: Megabases; MRCA: Most recent common ancestor; mya: Million years ago.

## Competing interests

The authors declare that they have no competing interests.

## Authors’ contributions

EBS and PGW conceived and drafted the paper; all other authors edited, contributed comments to, and read and approved the final manuscript.
